# Freiburg Neuropathology Case Conference: Recurrent Speech Arrest, Neologistic Jargon Aphasia, and Impaired Memory Function in a 39-year-old Patient

**DOI:** 10.1007/s00062-023-01335-6

**Published:** 2023-07-18

**Authors:** C. Storz, R. Sankowski, R. Roelz, M. Prinz, H. Urbach, D. Erny, C. A. Taschner

**Affiliations:** 1 Department of Neuroradiology, Medical Centre—University of Freiburg, Breisacherstraße 64, 79106 Freiburg, Germany; 2 Department of Neuropathology, Medical Centre—University of Freiburg, Freiburg, Germany; 3 Department of Neurosurgery, Medical Centre—University of Freiburg, Freiburg, Germany; 4 Faculty of Medicine, University of Freiburg, Freiburg, Germany

**Keywords:** Oligodendroglioma, Pleomorphic xanthoastrocytoma, Ganglioglioma, Angiocentric glioma, Polymorphous low-grade neuroepithelial tumor of the young

## Case Report

A 39-year-old man reported that he had experienced several short episodes of speech arrest and neologistic jargon aphasia in the last 6 months. He further described a noticeable limitation of his memory function. Convulsive seizures or loss of consciousness had not occurred. The patient presented without focal neurological deficits, speech disorders or apparent cognitive impairment. Cranial magnetic resonance imaging (MRI) revealed a left temporal lesion. Further epilepsy diagnostics were not performed in view of the clear causal relationship with the lesion.

The multidisciplinary tumor committee recommended total resection of the left temporal lesion because a high-grade glioma was suspected. A temporal craniotomy was performed. The left temporopolar cortex overlying the tumor was removed. It was a well-demarcated and relatively solid tumor, and these features allowed uncomplicated circular resection along the anatomical tumor borders. Most importantly, dissection against the basal ganglia, usually the most challenging step in the resection of temporal gliomas due to the lack of clear anatomical boundaries, was simple given the clear tumor margins. In summary, the microscopic aspect of the tumor was atypical of a high-grade glioma and more closely resembled a brain metastasis. The intraoperative histopathology, however, suggested a malignant glioma.

Postoperative MRI showed a complete resection of the contrast enhancing tumor. The patient recovered from surgery without new neurological deficits. An initial histological diagnosis of glioblastoma World Health Organization (WHO) grade IV, isocitrate dehydrogenase (IDH) wild type was made. Chemoradiotherapy with 54 Gy to the tumor region and six cycles of temozolomide were administered. The patient returned to full-time employment and reported no recurrence of speech disturbances or memory impairment.

Ultimately, the diagnosis was reversed from a glioblastoma WHO grade IV glioma to the final histological diagnosis based on the molecular characteristics of the tumor. The therapy had been adapted accordingly, and the further course of the disease was monitored by MRI. No recurrence was observed at 6 months after surgery.

## Imaging

**Fig. 1:**
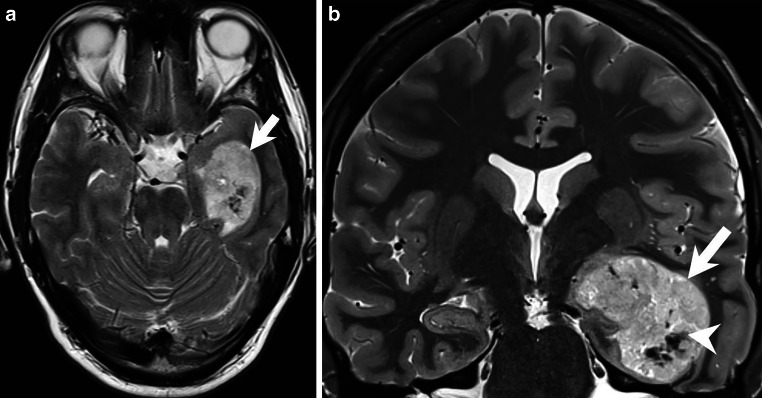
Axial (**a**) and coronal (**b**) T2-weighted images showing a clearly demarcated, intra-axial and space-occupying lesion in the left temporal lobe (*arrows*). The lesion had a relatively homogeneous hyperintense signal with circumscribed areas of signal loss in the lower parts of the tumor (**b**, *arrowhead*)

**Fig. 2:**
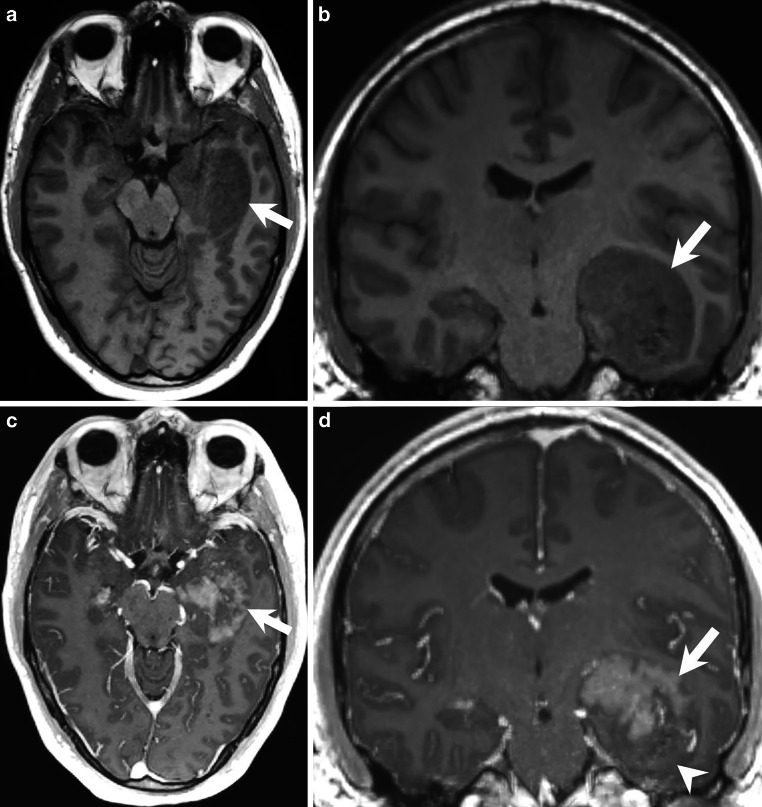
On axial (**a**) and coronal (**b**) T1-weighted images, the lesion appeared homogeneously hypointense (**a,** **b**, *arrows*). On axial (**c**) and coronal (**d**) T1-weighted images taken after administration of gadolinium (Gd), the lesion showed large areas, predominantly in the upper part of the lesion, with clear and homogeneous but not pronounced enhancement (**c,** **d**, *arrows*). In particular, the lower part of the lesion showed no contrast enhancement (**d**, *arrowhead*)

**Fig. 3:**
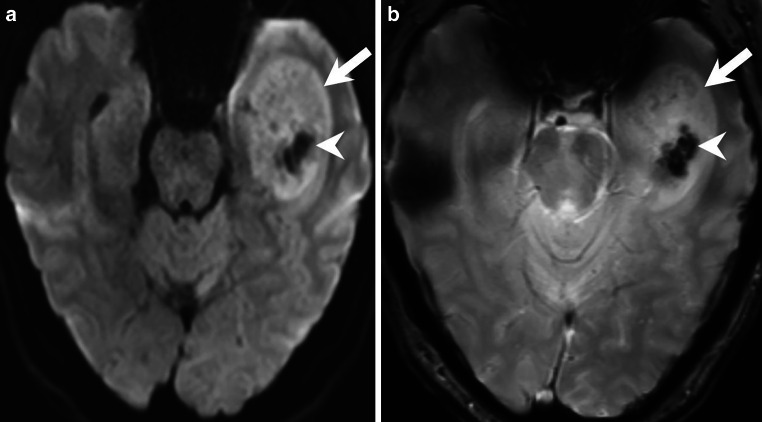
On axial diffusion-weighted images (b-value: 1000, **a**), the lesion showed signs of slightly restricted diffusion (*arrow*). Note the central and well-demarcated signal loss (**a**, *arrowhead*). On axial T2*-weighted images (**b**), this area within the lesion (**b**, *arrow*) also appeared homogeneously hypointense (**b**, *arrowhead*). This area could correspond to either local calcification or intralesional hemorrhage. Based on the MR images, we could not reliably distinguish the two. Unfortunately, we did not perform a preoperative computed tomography (CT) scan

Magnetic resonance imaging (MRI) showed a space-occupying intra-axial lesion of the left temporal lobe (Figs. 1, 2 and 3). On the T2-weighted images, the lesion appeared clearly demarcated and showed a predominantly hyperintense signal (Fig. 1a, b, arrow). In the center there were areas of homogeneous hypointense signal (Fig. 1b, arrowhead). On T1-weighted images, the lesion appeared hypointense (Fig. 2a, b, arrow). After administration of gadolinium (Gd), the lesion showed large areas, predominantly in the upper part of the lesion, with clear and homogeneous but not pronounced enhancement (Fig. 2c, d, *arrow*). Especially the lower part of the lesion showed no contrast enhancement (Fig. 2d, *arrowhead*). On diffusion-weighted MR images (b-value: 1000), the lesion showed signs of restricted diffusion (Fig. 3a, *arrow*). Note the central and well-demarcated signal loss (Fig. 3a, *arrowhead*). This area within the lesion (Fig. 3b, *arrow*) also appeared hypointense on T2*-weighted images (Fig. 3b, *arrowhead*). This area may correspond to either local calcification or intralesional hemorrhage. We could not reliably distinguish the two on the basis of the MR images. Unfortunately, we did not perform a preoperative computed tomography (CT) scan.

## Differential Diagnosis

### Glioblastoma

Glioblastomas are high-grade, fast-growing, diffusely infiltrating astrocytomas, classified as CNS WHO grade IV neoplasms, with a poor prognosis [1, 2]. Glioblastomas are the most frequent adult primary CNS tumors, commonly arising supratentorially in adults with a mean age of 64 years and a preponderance in men [3]. Due to the aggressive and rapid enlargement, glioblastomas are characterized by neovascularization and necrosis, surrounded by adjacent tumor infiltration and vasogenic edema [4]. On MRI, glioblastomas typically reveal a thick irregular enhancing margin with a central, partially hemorrhagic necrotic core, surrounded by large fluid-attenuated inversion recovery (FLAIR) hyperintense edema and diffuse non-enhancing tumor infiltration [4]. According to the frequency of occurrence of glioblastomas in adults in combination with the imaging features in the present case with a large irregularly enhancing mass, a glioblastoma was considered as a valid differential diagnosis.

### Oligodendroglioma

Oligodendrogliomas are diffuse infiltrating gliomas classified as WHO CNS grade II–III neoplasms, with the molecular hallmark feature of IDH mutation and 1p19q codeletion [1]. Accounting for approximately 5–25% of all gliomas, oligodendrogliomas are usually seen in middle-aged adults with a slight male predilection [5, 6]. Oligodendrogliomas usually arise in the cerebral hemispheres, especially in the frontal lobe, most commonly in a cortical-subcortical location [5]. In 70–90% the tumor characteristically contains calcification, often in a peripherally distributed gyriform configuration [5]. Furthermore, remodelling of the adjacent calvarium is a characteristic finding in oligodendrogliomas [5]. On MRI, oligodendrogliomas typically appear hypointense in T1-weighted, and hyperintense in T2-weighted sequences, whereas contrast enhancement can show a broad variety from nonenhancement to diffuse enhancement to rim enhancement (in up to 50% of cases) [5, 6]. In a few cases, oligodendrogliomas can contain focal hemorrhage or cystic elements of the tumor [6].

Given the age of the patient in the present case as well as the calcified alterations, an oligodendroglioma has to be considered as differential diagnosis, although the temporal and mostly subcortical location as well as the relatively well-circumscribed and enhancing appearance is not typical for an oligodendroglioma.

### Dysembryoplastic Neuroepithelial Tumor

Dysembryoplastic neuroepithelial tumors (DNET) are slowly growing, well-circumscribed, cortically based tumors, classified as CNS WHO grade I neoplasms [1, 7, 8]. Overall, DNETs are rare entities, occurring mostly in children and young adults, often associated with epilepsy [1, 8]. The vast majority arise supratentorially in the temporal lobe, with a typically multifocal cystic “bubbly” appearance with low signal in T1-weighted and high signal in T2-weighted sequences and low cellular density [7–9]. DNETs can be associated with cortical abnormalities such as focal cortical dysplasia [8]. Contrast enhancement is rather rare in DNET, as only 20% display postcontrast enhancement of the lesion. Hemorrhage or calcification is rarely seen [7–9].

Due to the calcification and inhomogeneous enhancement of the lesion in the present case as well as the missing “bubbly” or cystic component, the diagnosis of a DNET is less likely.

### Pleomorphic Xanthoastrocytoma

Pleomorphic xanthoastrocytomas (PXA) are rare astrocytic tumors, classified as WHO CNS grade II–III tumors, depending on the histological features [1]. Usually seen in children and young adults, PXAs typically arise supratentorially, classically in the temporal lobe, and are thus often associated with temporal lobe epilepsy [1, 10]. Characteristically, PXAs appear as superficial cortical tumors with a solid enhancing nodule and a peripheral cystic component [10, 11]. Due to the superficial location, leptomeningeal involvement with a reactive leptomeningeal enhancement exhibiting a dura tail sign is common [10, 11]. Owing to its slow growth and the superficial location, thinning of adjacent calvarium can rarely be found [10, 11].

Due to the described imaging features of PXAs, the diagnosis is less likely in the present case, as a solid enhancing nodule with cystic component and leptomeningeal involvement are absent; however, due to the occurrence in the temporal lobe, the diagnosis of PXA must be considered.

### Ganglioglioma

Gangliogliomas are rare tumors of the central nervous system (CNS), usually low grade, and reveal a combination of neuronal and glial cell elements with a variable morphological spectrum [1, 12, 13]. Malignant transformation of the glial components is very rare [13]. Although gangliogliomas can occur in all parts of the CNS, they typically arise in the temporal lobe and represent the most common tumor entity in children with epilepsy [12, 13]. Gangliogliomas reveal various imaging patterns, often occurring with an enhancing nodule with a cystic component [12–14]; however, they can also appear as solid tumors with variable contrast enhancement or as a diffuse infiltrating poorly delineated mass [12–14]. Calcifications can be frequently detected [12–14].

In the present case the tumor is characterized by a solid mass in the temporal lobe with calcified alterations and inhomogeneous enhancement, thus, and due to the various appearance of gangliogliomas, this differential diagnosis has to be taken into account.

### Angiocentric Glioma

An angiocentric glioma is a rare low-grade diffuse tumor entity with angiocentric pattern, usually affecting children and young adults [1]. They typically arise cortically or subcortically in the parietal or temporal lobe. On MRI they characteristically appear as a well-delineated T2/FLAIR hyperintense lesion, occasionally with a T1 hyperintense rim and a stalk-like extension towards the ventricle without contrast enhancement [15–17]. Due to the missing stalk-like appearance and the clearly definable contrast enhancement, this differential diagnosis is less likely in the present case.

### Diffuse Astrocytoma

Diffuse astrocytomas are low-grade diffusely growing neoplasms, MYB-altered or MYBL1-altered, and commonly arise within the cortex or subcortex of the temporal and frontal lobes [15, 16, 18]. Usually, diffuse astrocytomas are well-delineated T2-hyperintense lesions without contrast enhancement [16, 18]. Especially in adolescents and young adults, diffuse astrocytomas, MYB-altered or MYBL1-altered, have to be distinguished from IDH-wildtype and IDH-mutated diffuse gliomas. These neoplasms reveal similar histopathologic features but express more aggressive characteristics resulting in higher CNS WHO grades, which is reflected in ill-delineated masses and more frequent contrast enhancement in imaging [16].

### Polymorphous Low-Grade Neuroepithelial Tumor of the Young

Polymorphous low-grade neuroepithelial tumors of the young (PLNTY) were firstly described in 2017 by Huse et al. [19]. PLNTYs are diffuse low-grade gliomas, classified as CNS WHO grade I neoplasms, mostly arising in children and young adults, with an age range of 5–32 years (median 16 years) [20, 21]. One case of malignant transformation in a recurrent PLNTY has so far been reported, suggesting a broad molecular spectrum [22]. The PLNTYs are mostly cortically and/or subcortically located and frequently arise in the temporal lobe [19, 20, 23, 24]. These lesions mostly have mixed FLAIR hyperintense solid and cystic components with oligodendroglioma-like cellular elements and typically no mass effect [19, 20, 23, 24]. The imaging hallmark of PLNTYs are centrally located dense calcifications, often described as a salt and pepper sign [19, 20, 24]. Most PLNTYs show no enhancement in contrast enhanced T1-weighted sequences, although a patchy mild enhancement is found in a minority of the cases [20, 23]. In the present case, the subcortical/cortical temporal location and centrally distributed calcification of the tumor raise suspicion of PLNTY, while the relatively atypical contrast enhancement, space-occupying features and the patient’s age should prompt consideration of other differential diagnoses.

## Histology and Immunohistochemistry

**Fig. 4:**
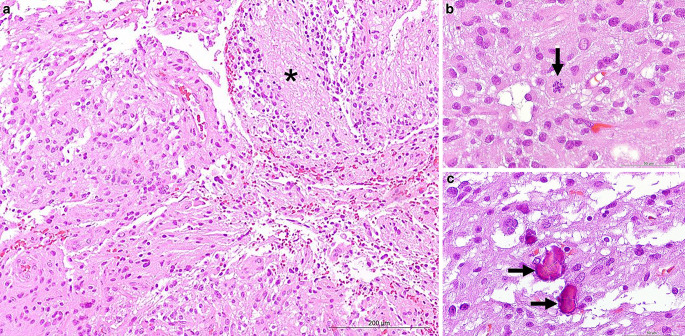
Hematoxylin-eosin (*H&E*) stained sections (**a–c**) show a glial tumor with necrotic areas (**a**, *asterisk*), and mitotic spindles indicating elevated proliferation (**b**, *arrow*). In addition, calcifications were present (**c**, *arrows*). Size bars: **a** 200 µm; **b,** **c** 50 µm

**Fig. 5:**
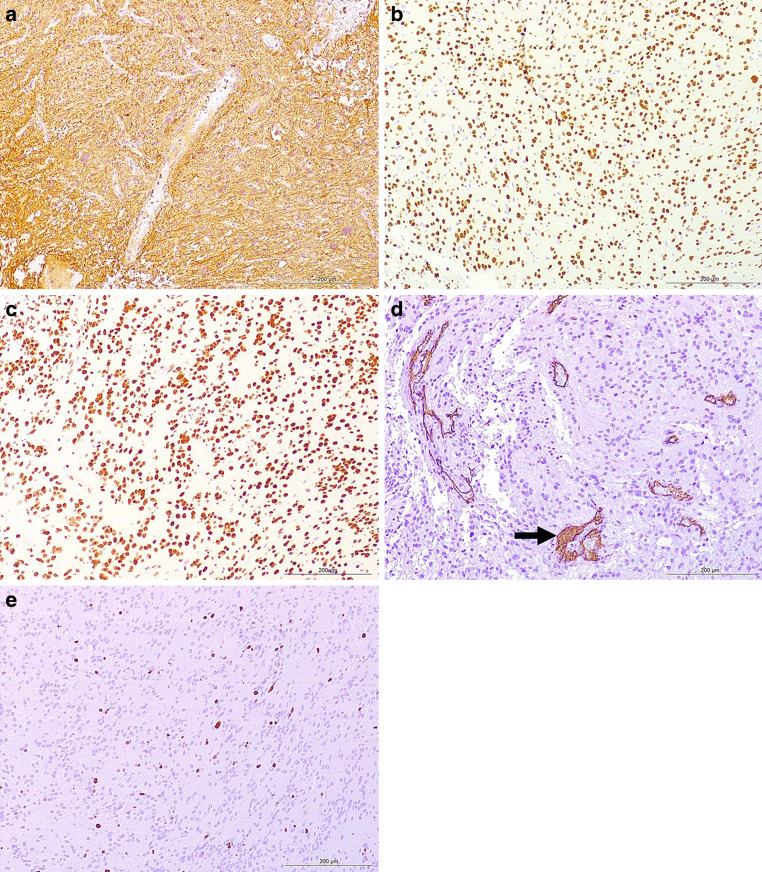
Immunohistochemistry shows glial fibrillary acidic protein (GFAP, **a**) and oligodendrocyte transcription factor (OLIG2, **b**) positive tumor cells, indicating glial differentiation of the tumor. The nuclear ATRX expression is retained suggesting the absence of e.g., mutations, deletions or fusions of the *ATRX* gene (**c**). Microvasculature proliferation is depicted by CD34 immunohistochemistry (*arrow*) (**d**). The proliferation index is increased tagging up to 10% of tumor cells, depicted by MIB1 immunohistochemistry (**e**). Images were taken at 100 × magnification and the scale bars represent 200 µm

Hematoxylin and eosin staining of the formaldehyde-fixed and paraffin-embedded biopsy material revealed a pleomorphic glial tumor with necroses and microvascular proliferations (Fig. 4a), scattered mitotic spindles (Fig. 4b) and calcifications (Fig. 4c). Immunohistochemistry showed positivity for glial fibrillary acidic protein (GFAP), oligodendrocyte transcription factor (Olig2), and a retained nuclear expression of ATRX (Fig. 5a–e). CD34 was negative except for individual tumor cells (Fig. 5d). The proliferation marker Ki-67 was found to be positive in up to 10% of the tumor nuclei (Fig. 5e). Based on these features the initial diagnosis was glioblastoma WHO grade IV

Differential diagnoses included high-grade gliomas, astrocytoma, oligodendroglioma, diffuse pediatric-type high-grade glioma, H3-wildtype and IDH-wildtype, diffuse hemispheric glioma, H3 G34-mutant, and some rare neuroectodermal tumors with a diffuse growth pattern. These differential diagnoses are characterized by pleomorphic tumor cells with broad morphological overlaps. Therefore, additional molecular testing was required [1]. Methylation analysis is a fast and cost-efficient way to molecularly distinguish these entities [25]. Glioblastomas are characterized by a distinct methylation and genomic instability with a typical combination of a chromosome 7 gain and a chromosome 10 loss [1]. Astrocytomas and oligodendrogliomas are defined as having pathological hotspot isocitrate dehydrogenase (IDH) mutations [1]. Additionally, oligodendrogliomas show a combined loss of the chromosome arms 1p/19q. Diffuse hemispheric gliomas, *H3 G34* mutant, have mutations of the *histone H3-3A* gene and a characteristic methylation profile [1]. Diffuse pediatric-type high-grade glioma, H3-wildtype and IDH-wildtype, lack these mutations but have a characteristic methylation profile [1]. Additionally, these tumors are frequently associated with previous brain irradiation and constitutional mismatch repair deficiency syndromes [26]. As the morphological appearance of these tumors is unspecific, molecular pathology was initiated.

Molecular testing showed a TP53 loss of function mutation, a common finding in high-grade gliomas. Wild-type alleles were found in the *IDH* and *histone H3* genes. The copy number profile revealed signs of genomic instability with gains of genomic material on several chromosomes including chromosome 7 and chromothripsis on chromosome 10. A combined loss of the chromosome arms 1p/19q was not found. Next, an 850k methylation array analysis was performed. Classification with the Heidelberg brain tumor classifier version 12.5 failed to generate a match with the established methylation classes [25]. Unexpectedly, the highest scores were associated with methylation classes featuring low-grade neuroectodermal tumors, including ganglioglioma and polymorphous low-grade neuroepithelial tumor of the young (PLNTY). The RNA sequencing identified a FGFR2::CTNNA3 fusion. This molecular aberration has so far only been observed in PLNTYs [27].

## Diagnosis

### Polymorphous Low-Grade Neuroepithelial Tumor of the Young (PLNTY), CNS WHO Grade 1 with Features of a Malignant Transformation

In 2016 Huse et al. reported a new type of epileptogenic tumor and named the entity as PLNTY [19]. PLNTYs have been described in patients between 4 and 57 years of age with the majority of patients between 10 and 30 years old [19, 20, 28, 29]. Clinically, these tumors are often associated with refractory epilepsy and other neurological symptoms including headache and dizziness [19, 28–31]. It is notable for the presence of oligodendroglioma-like cellular components, CD34 immunopositivity, and an association with cortical dysplasia [32]. Molecularly, PLNTYs show MAPK pathway-activating aberrations, including somatic mutation, duplications, and fusions of the *BRAF, FGFR2, FGFR3*, and, rarely, *NTRK* genes [19, 20, 28]. Notably, the FGFR2::CTNNA3 fusion identified above has so far only been reported in PLNTYs [27]. Conversely, IDH mutations or a combined loss of the chromosome arms 1p/19q are not observed.

Due to a relative scarcity of reported cases, prognostic evidence on PLNTYs is limited to date. Accurate diagnosis is critical in predicting clinical behavior and developing a treatment plan. In a published case series the majority of PLNTYs showed a clinical behavior consistent with CNS WHO grade 1 tumors with rare recurrences after gross total resection [19]. Given the overlap between the clinical, radiologic, and histopathologic presentation of this tumors with others, specifically oligodendroglioma, the potential for misdiagnosis is significant. PLNTY is associated with an indolent course and seizure control can typically be attained with gross total resection. Oligodendroglioma is associated with a lower rate of epilepsy and a worse clinical prognosis [32]. A malignant transformation with high-grade histological features was reported in one case featuring an FGFR2::CTNNA3 fusion and somatic mutations of the *TP53, ATRX, PTEN*, and *TEK* genes [22].

In summary, the present case is diagnosed as PLNTY, CNS WHO grade 1; however, the pleomorphic histopathology and the identified TP53 loss of function mutation suggest that the tumor has undergone a malignant transformation warranting frequent follow-up controls.
